# *Nicotianabenthamiana*, a Surrogate Host to Study Novel Virulence Mechanisms of Gram-Positive Bacteria, *Clavibacter michiganensis*, and *C. capsici* in Plants

**DOI:** 10.3389/fpls.2022.876971

**Published:** 2022-05-10

**Authors:** In Woong Park, In Sun Hwang, Eom-Ji Oh, Choon-Tak Kwon, Chang-Sik Oh

**Affiliations:** ^1^Department of Horticultural Biotechnology, Kyung Hee University, Yongin, South Korea; ^2^Graduate School of Green-Bio Science, Kyung Hee University, Yongin, South Korea

**Keywords:** *Clavibacter* *michiganensis*, *Nicotiana benthamiana*, surrogate host, virulence factors, virulence mechanisms

## Abstract

*Clavibacter michiganensis* is a Gram-positive bacterium that causes bacterial canker and wilting in host plants like tomato. Two major virulence genes encoding a cellulase (*celA*) and a putative serine protease (*pat-1*) have been reported. Here we show that *Nicotiana benthamiana*, a commonly used model plant for studying molecular plant–pathogen interactions, is a surrogate host of *C. michiganensis* and *C. capsici*. When a low concentration of two *Clavibacter* species, *C*. *michiganensis* and *C*. *capsici*, were infiltrated into *N. benthamiana* leaves, they caused blister-like lesions closely associated with cell death and the generation of reactive oxygen species and proliferated significantly like a pathogenic bacterium. By contrast, they did not cause any disease symptoms in *N. tabacum* leaves. The *celA* and *pat-1* mutants of *C*. *michiganensis* still caused blister-like lesions and cankers like the wild-type strain. When a high concentration of two *Clavibacter* species and two mutant strains were infiltrated into *N. benthamiana* leaves, all of them caused strong and rapid necrosis. However, only *C. michiganensis* strains, including the *celA* and *pat-1* mutants, caused wilting symptoms when it was injected into stems. When two *Clavibacter* species and two mutants were infiltrated into *N. tabacum* leaves at the high concentration, they (except for the *pat-1* mutant) caused a strong hypersensitive response. These results indicate that *C. michiganensis* causes blister-like lesions, canker, and wilting in *N. benthamiana*, and *celA* and *pat-1* genes are not necessary for the development of these symptoms. Overall, *N. benthamiana* is a surrogate host of *Clavibacter* species, and their novel virulence factors are responsible for disease development in this plant.

## Introduction

*Nicotiana benthamiana* is commonly used as a model plant in many studies of plant–microbe interactions (Goodin et al., 2008; Bombarely et al., 2012). This plant has many benefits: a rapid life cycle, ease of seed harvest, and relatively small plant size (which precludes any large space requirement) (Davarpanah et al., 2009). *N*. *benthamiana* has been shown to be susceptible *via* artificial inoculation against many kinds of pathogenic microorganisms, such as Gram-negative bacteria, fungi, oomycetes, and viruses. Thus, this plant has been used in studies of various molecular interactions with microbes mostly *via* virus-induced gene silencing and transient expression assay (Liu et al., 2002; Tran et al., 2016). However, pathogenic interactions between *N*. *benthamiana* and Gram-positive bacteria, including *Clavibacter* species, have not been well studied.

The genus *Clavibacter* belongs to the family *Microbacteriaceae* within Actinobacteria and includes seven Gram-positive and plant-pathogenic species (Li et al., 2018). *C*. *michiganensis* is an important bacterial pathogen of the tomato plant (*Solanum lycopersicum*). It can be transmitted by, and survive in, contaminated seeds and plant debris of tomatoes (de León et al., 2011; Tancos et al., 2013). When *C*. *michiganensis* invades the tomato plant through wounded stems, roots, leaves, and natural openings, it moves to plant xylem, eventually impairing xylem vessels and causing wilting. Moreover, it causes various disease symptoms in tomato, such as cankers on stems, blister lesions on leaves, bird’s eye lesions on fruits, and plant death (Medina-Mora et al., 2001; Sen et al., 2015; Chalupowicz et al., 2017). *C. capsici* causes bacterial canker in stems, fruits, and leaves of pepper plants. This bacterium was re-classified from *C. michiganensis* by biochemical, physiological, and phylogenetic analyses (Oh et al., 2016).

A few virulence factors of *C*. *michiganensis* have been reported. A pathogenicity island (PAI), a subset of genomic islands, is present on the chromosome (Gartemann et al., 2008). Approximately 129 kb of PAI carries the *chp*/*tomA* region, further subdivided into two subregions, namely, the *chp* and the *tomA* subregions. These subregions carry genes encoding putative serine proteases, such as *chpC* and *chpG* (Stork et al., 2008), and tomatinase (Kaup et al., 2005; Eichenlaub and Gartemann, 2011), respectively. *C*. *michiganensis* also harbors two major virulence genes in two plasmids, namely, pCM1 and pCM2; each plasmid carries *celA* and *pat-1* genes, which encode cellulase (endo-β-1,4-glucanase) and a putative serine protease, respectively (Dreier et al., 1997; Jahr et al., 2000; Hwang et al., 2019). In a previous study, each plasmid-cured mutant strain showed reduced and/or lost pathogenicity, compared with the wild-type (WT) strain, whereas each maintained its proliferation ability (Chalupowicz et al., 2012), indicating that these two genes in plasmids are critical for virulence in tomatoes. In the case of *C. capsici*, its virulence factors have not been well-studied yet, but some *chp* genes, such as *chpG* and *chpE* genes, present in pCM1_Cc_ plasmid have been reported (Hwang et al., 2018, 2020).

*C*. *michiganensis* has been shown to naturally infect other *Solanum* plants, such as eggplant (*S*. *melongena*), potato (*S*. *tuberosum*), and black nightshade (*S*. *nigrum*) (Bradbury, 1986; Ignatov et al., 2019), besides its well-known tomato host plant. Moreover, plants in other genera, such as *Datura*, *Hyoscyamus*, and *Physalis*, can be infected; disease symptoms can also develop *via* artificial inoculation with *C*. *michiganensis* (Thyr et al., 1975; Eichenlaub et al., 2007). After inoculation with *C. michiganensis*, *N. benthamiana* plant displayed canker symptoms on its stems (Balaji et al., 2011), indicating this plant’s potential as a surrogate host for *C. michiganensis*. Artificial or surrogate host plants displayed disease symptoms *via* artificial inoculation of the pathogens. For example, the model plant *Arabidopsis thaliana* is routinely used as a surrogate host plant to study molecular plant interactions with *Pseudomonas syringae* pv. *tomato* DC3000, which causes bacterial speck disease on tomato (Xin and He, 2013). *N*. *benthamiana* has been reported to be a surrogate host of plant pathogens including *Acidovorax citrulli*, which causes bacterial fruit blotch in cucurbits. This bacterium caused disease symptoms of water-soaking-like cell death, but not hypersensitive response (HR), on *N*. *benthamiana* leaves *via* syringe infiltration (Traore et al., 2019).

In this study, we conducted several experiments to investigate the pathogenic interactions between *N*. *benthamiana* and the representative species of genus *Clavibacter*, that is, *C*. *michiganensis* and *C*. *capsici*. We also examined whether the known important virulence genes of *C*. *michiganensis* in tomato are responsible for the development of disease symptoms in *N*. *benthamiana*. We found that *N*. *benthamiana* is a surrogate host plant of *C*. *michiganensis* and *C. capsici*, and different virulence genes of *C*. *michiganensis* are required for the development of blister-like lesions, rapid necrosis, canker, and wilting.

## Materials and Methods

### Plant Materials and Growth Conditions

Two tobacco species, *N*. *benthamiana* and *N*. *tabacum*, were grown in 32 cell seedling trays filled with sterile commercial soil (Baroker, Seoul Bio Co., Ltd., Eumseong, Korea) in a growth chamber at 26°C with a photoperiod of 14 h of light and 10 h of darkness. Five- to six-week-old *N*. *benthamiana* plants were used for virulence tests *via* syringe infiltration, stem inoculation, and spray inoculation. *N*. *benthamiana* plants at the four- to six-leaf stage (around 3 weeks old) were used for virulence tests *via* root-dip inoculation. Seven- to eight-week-old *N*. *tabacum* plants were used for virulence and bacterial growth tests on the leaves *via* syringe infiltration.

### Bacterial Strains and Growth Conditions

*C*. *michiganensis* type strain LMG7333^T^ (Hwang et al., 2019), *C*. *capsici* type strain PF008^T^ (Oh et al., 2016), and *A*. *citrulli* strain Ac8 (Song et al., 2020) were cultured at 26°C for 48 h in the King’s B medium (20 g/l of protease peptone number 3, 1.5 g/l of K_2_HPO_4_, 6 ml/l of 1 M MgSO_4_, and 16 ml/l of 50% glycerol). Tn::*celA* (Hwang et al., 2019) and Tn::*pat-1* mutants (Hwang et al., 2022) of *C*. *michiganensis* LMG7333^T^ were streaked onto the King’s B medium with 10 mg/l of chloramphenicol at 26°C for 48 h.

### Virulence Assay in *Nicotiana* Plants

For the leaf infiltration, five- to six-week-old *N*. *benthamiana* and seven- to eight-week-old *N*. *tabacum* plant leaves were infiltrated with either sterilized distilled water (mock) or 5×10^4^ CFU/ml bacterial suspension or approximately 10^8^ CFU/ml (OD_600_ = 0.1) bacterial suspension using a needleless syringe; at least three leaves were infiltrated per treatment. All inoculated plants were placed in a growth chamber at 26°C and 50% humidity. The development of blister-like symptoms and necrosis was observed for 8 days and 48 hours, respectively. This assay was repeated at least three times.

For stem inoculation, five- to six-week-old *N*. *benthamiana* plants were injected with 20 ul of 10 mM MgCl_2_ (mock) or 10^8^ CFU/ml (OD_600_ = 0.1) bacterial suspension after wounding the stems with a pipette tip. All inoculated plants were placed in a growth chamber at 26°C and 50% humidity. Wilting development was observed for 3 weeks. Wilted leaves were counted and compared with the number of fully grown leaves above the inoculation site. The ratio between wilted leaves and fully grown leaves was calculated by percentage. Bacterial canker symptoms on stem inoculation sites were observed at 0, 5, 10, and 15 days after inoculation (dai), and their sizes were measured as lengths of their longitudinal crack using a ruler. This assay was repeated at least three times.

For the root-dip inoculation, *N*. *benthamiana* plants in the four- to six-leaf stage were pulled out and dipped in the bacterial suspension (approximately 10^9^ CFU/ml, OD_600_ = 2.0) for 30 or 40 min, depending on plant size. Then, the inoculated plants were replanted into soil in a new tray. All inoculated plants were placed in a growth chamber at 26°C and 50% humidity. Wilting development was observed for 3 weeks. Root-inoculated *N*. *benthamiana* plants were analyzed by the number of plants displaying wilting using five categories defining the severity of wilting symptoms: (1) 0 = no visible wilting; (2) 1 = 1–25% wilting symptoms; (3) 2 = 26–50% wilting symptoms; (4) 3 = 51–75% wilting symptoms; (5) 4 = 76–100% wilting symptoms or dead at around 18 dai. The disease index used in this study was described in previous research (Shinohara et al., 2005). This assay was repeated at least three times.

For the spray inoculation, *N*. *benthamiana* plants were sprayed with 50 ml of bacterial suspensions (approximately 5×10^6^ CFU/ml, OD_600_ = 0.005 and 10^8^ CFU/ml, OD_600_ = 0.1) containing 0.02% Silwet L−77 (MOMENTIVE Co., Ltd., Seoul, Korea). Wilting development was observed for more than 3 weeks. All inoculated plants were placed in a growth chamber at 26°C and 50% humidity. This assay was repeated twice.

### Measurement of Bacterial Growth in *Nicotiana* Leaves

To measure the bacterial growth inside both *N*. *benthamiana* and *N*. *tabacum* leaves after syringe infiltration with 5×10^4^ CFU/ml bacterial suspension, three-leaf disks (1 cm in diameter) were collected from the infiltrated leaves of at least three different plants using a cork borer at 0, 2, 4, 6, and 8 dai. The collected leaf disks were washed with 70% ethanol for 30 s for surface sterilization and were rinsed with sterilized distilled water twice. Next, washed leaf disc samples were ground by vortexing them in 2 ml microtubes with two iron beads and 1 ml of sterilized distilled water. The homogenate was serially diluted and put onto a KB agar plate. The numbers of colony-forming units (CFU) were calculated after 48–72 h at 26°C.

### Ion Conductivity Measurement in *Nicotiana* Leaves

For quantification of necrosis in *N*. *benthamiana* leaves, ion conductivity was measured as described in the previous study (Choi et al., 2012). At least 4 leaves selected from different plants were infiltrated with 10^8^ CFU/ml (OD_600_ = 0.1) bacterial suspension using needleless syringe. After drying drenched leaves completely for 60 or 90 min, six-leaf disks (1 cm in diameter) were taken from each dried leaf using a cork borer. The leaf disks were moved to a 50 ml snap tube with 25 ml of sterilized distilled water to remove the initially leaked ion until 3 h after inoculation (hai). Then, water inside a 50 ml snap tube was removed completely, except for leaf disks, and 25 ml of sterilized distilled water was added to the snap tube again. Ion conductivity was measured using a conductivity meter (Acorn CON6 portable conductivity meter, Oakton Instrument, Vernon Hills, IL, USA) at 3, 12, 24, 36, and 48 hai. The snap tubes with leaf disks were incubated on the shaker at 60 rpm. Four of 50 ml snap tubes with six-leaf disks and 25 ml sterilized distilled water were used for each bacterial strain. The experiments were repeated at least twice.

To quantify the HR in *N*. *tabacum* leaves, six-leaf disks (1 cm in diameter) were collected from the infiltrated leaves with each bacterial strain at 3, 9, 15, and 21 hai. Leaf disks were moved to a 50 ml snap tube with 25 ml of sterilized distilled water and were washed for an hour. Then, the same volume of water was added to the snap tube. Next, those tubes were incubated for an hour in the shaking incubator at 26°C and 180 rpm, and ion conductivity was measured using a conductivity meter. The experiments were repeated at least twice.

### Trypan Blue Staining in *N. benthamiana* Leaves

The trypan blue staining procedure was modified from a previous study (Guo et al., 2019). Briefly, the leaf disks (1 cm in diameter) from leaves of at least 3 different *N*. *benthamiana* plants infiltrated using a syringe with 5×10^4^ CFU/ml of *C*. *michiganensis* LMG7333^T^ and *C*. *capsici* PF008^T^ were taken using a cork borer at 0, 3, 5, and 7 dai. Sterilized distilled water was used for mock treatment. Leaf disks were treated with trypan blue to stain damaged or dead cells. A 3 ml trypan blue solution (10 ml lactic acid, 10 g phenol, 10 ml glycerol, 10 ml distilled water, and 10 mg trypan blue) was added into a 5 ml tube with leaf disks. One day later, the trypan blue solution was exchanged with 4 ml of absolute ethanol to remove unstained trypan blue. After 1 day of ethanol bleaching, the absolute ethanol in the 5 ml tube was exchanged with 70% ethanol for storage until the time that leaf disks were observed using a microscope. Leaf disks were observed using an optical microscope (ECLIPSE E200LED MV R, Nikon Corporation, Tokyo, Japan) at a magnification x40.

### 3,3′-Diaminobenzidine Staining in *N. benthamiana* Leaves

The DAB staining method was followed and modified from a previous study (Shi et al., 2014). Briefly, leaf disks (1 cm in diameter) were collected from at least three different *N*. *benthamiana* plants infiltrated with 5×10^4^ CFU/ml of *C*. *michiganensis* LMG7333^T^ and *C*. *capsici* PF008^T^ using a cork borer at 0, 1, 3, 5, and 7 dai. Sterilized distilled water was used for mock treatment. The collected leaf disks were placed in the petri dish and stained with 10 ml of 0.1% DAB solution for 1 day. Next, DAB solution was removed from the petri dish, and 10 ml of absolute ethanol was added to the petri dish to bleach the leaf disk chlorophyll. After 1 day of ethanol bleaching, absolute ethanol in the petri dish was exchanged with 70% ethanol for storage until the time that leaf disks were observed using a microscope. All DAB-stained *N*. *benthamiana* leaf disks were observed using an optical microscope at a magnification x40.

### Detection of *Clavibacter* Bacteria in Plants *via* PCR Analysis

PCR analysis was conducted for the detection of *Clavibacter* bacteria in the plants after inoculation. PCR amplification proceeded according to the manufacturer’s manual for 2x Taqbasic PCR Master Mix 2 (Biofact, Daejeon, Korea). The total volume was 20 uL and consisted of 10 ul of 2X Taqbasic PCR Master Mix 2, 1 ul of template, 1ul of forward and reverse primers (10 pmol/ul), and 7 ul of sterilized distilled water.

Several specific primer pairs were used as follows. Primer pair CMR16S F (5′-gtgatgtcagagcttcctctggcggat-3) and CMR16S R (5′-gtacggctaccttgttacgacttagt-3′) were used for *C*. *michiganensis* LMG7333^T^ and *C*. *capsici* PF008^T^, because they specifically target the 16S-rRNA gene of *Clavibacter* species (Yim et al., 2012). For confirmation of *C*. *michiganensis* Tn::*celA* and Tn::*pat-1* mutant strains, the H1 (5′-atgacatttcgccaagttcgtgca-3′) and H2 (5′-tcagtgcacagggtagaagcg-3′) primer pair was used for *celA* gene. The EB15 (5′- actagtagaacgctccctgcggccttcg-3′) and EB16 (5′- aagcttacttgtcgtcatcgtctttgtagtcggaggtcgctaatatgtaatacggt-3′) primer pair was used for *pat-1* gene.

### Statistical Analysis

Bacterial growth was expressed as mean and standard deviations. The disease index of stem and root inoculation was calculated as the mean and standard error. Disease severity data were analyzed *via* non-parametric Kruskal–Wallis test (*p* < 0.05). Duncan’s multiple range test was conducted for statistical analysis of the parametric comparison between independent groups (*p* < 0.05). All experiments were repeated at least two or three times.

## Results

### Two *C.* Species Caused Blister-Like Lesions in Leaves of *N. benthamiana*, but Not *N. tabacum*

**Figure 1:**
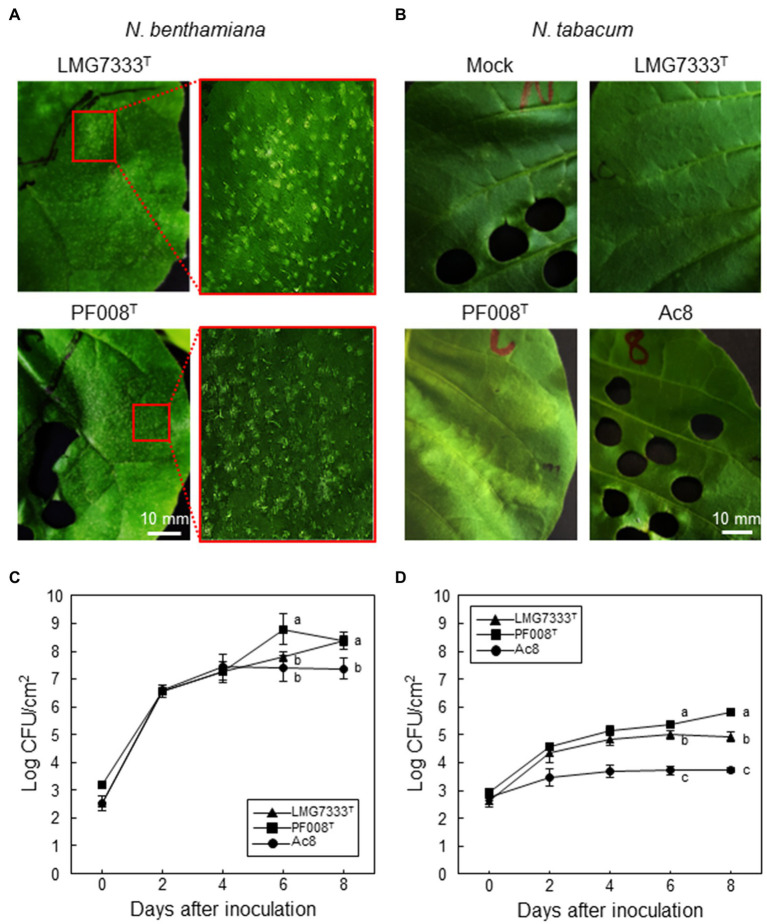
Disease symptoms and bacterial growth in *N*. *benthamiana* and *N*. *tabacum* leaves. Both *Clavibacter* species induced blister-like symptoms on *N*. *benthamiana* **(A)**, whereas no visible symptoms were induced on *N*. *tabacum* **(B)**, when 5×10^4^ CFU/ml of *C*. *michiganensis* LMG7333^T^ and *C*. *capsici* PF008^T^ bacterial suspensions were infiltrated. Photos were taken at 4 days after infiltration (dai). Mock, 10 mM MgCl_2_. Bacterial growth in *N*. *benthamiana* **(C)** and *N*. *tabacum* **(D)** leaves was measured at the indicated time points. The *A*. *citrulli* strain Ac8 was used as a positive control. An average and standard deviation (*n* = 3) of each bacterial titer are shown in the figures. The different letters at 6 and 8 dai in the graphs indicate a statistically significant difference analyzed *via* Duncan’s multiple range test (*p* < 0.05). Similar results were obtained from three independent assays. Scale bar = 10 mm.

To investigate whether Gram-positive *Clavibacter* bacteria can cause any disease symptoms in model plants of *Nicotiana* species, both *C*. *michiganensis* LMG7333^T^ and *C*. *capsici* PF008^T^ were infiltrated into leaves of two *Nicotiana* species, *N*. *benthamiana* and *N*. *tabacum*. *N*. *benthamiana* leaves showed white granular particles, called blister-like lesions, when 5×10^4^ CFU/ml of each bacterial species was infiltrated (Figure 1A). These lesions were generated on the leaf surface around 4 dai. The formation of blisters was originally reported in tomato, the host plant of *C*. *michiganensis* (Chalupowicz et al., 2017), and blister-like lesions on *N. benthamiana* appeared phenotypically similar to those in tomato leaves. Conversely, *N*. *tabacum* leaves did not display any disease symptoms by infiltration with the same titer of either *C*. *michiganensis* LMG7333^T^ or *C*. *capsici* PF008^T^ suspension (Figure 1B). In *N*. *benthamiana* leaves, the infiltrated bacterial pathogens grew significantly up to 10^6^-fold more than the initial inoculum until 8 dai (Figure 1C); an increase was more than that by the known pathogen *A. citrulli*. However, the bacterial titer of both *Clavibacter* species increased only 10^2^- to10^3^-fold more in *N*. *tabacum* leaves from the initial inoculum without any symptoms, and this increase was higher than that by *A. citrulli* (Figure 1D). Moreover, the bacterial titer of *C*. *capsici* was significantly higher than *C*. *michiganensis* (Figure 1D). These results indicate that *N. benthamiana* appears to be a surrogate host plant of two *Clavibacter* species, whereas *N. tabacum* appears to be a non-host plant.

### Blister-Like Lesions on *N. benthamiana* Leaves Are Closely Associated With Cell Death and the Generation of Reactive Oxygen Species

**Figure 2:**
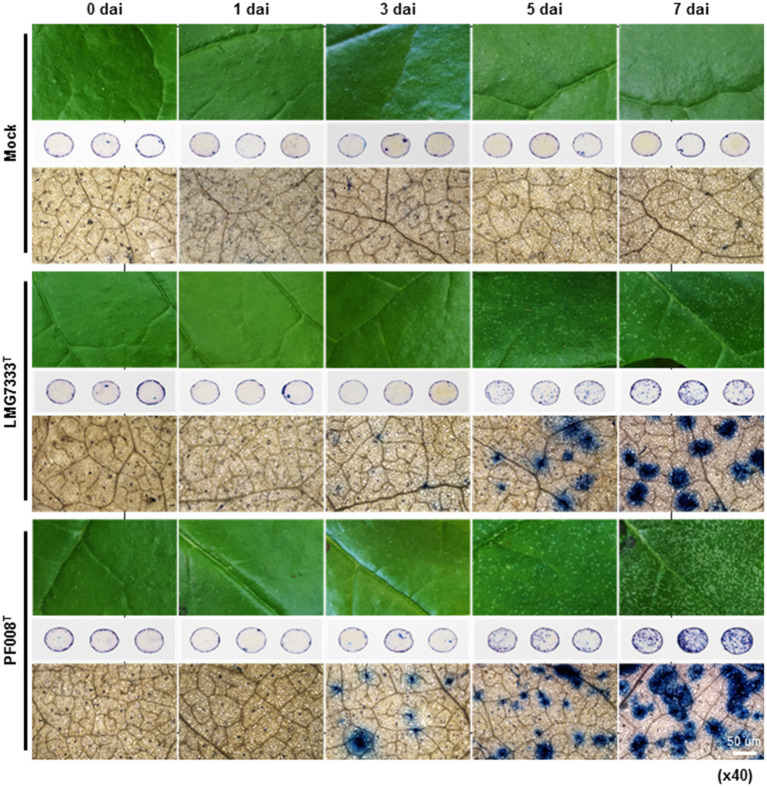
Trypan blue staining of *N*. *benthamiana* leaves showing blister-like symptoms, after infiltration with 5×10^4^ CFU/ml bacterial suspensions of *C*. *michiganensis* LMG7333^T^ and *C*. *capsici* PF008^T^. Three-leaf disks (1 cm in diameter) per treatment were collected for trypan blue staining at 0, 1, 3, 5, and 7 days after inoculation (dai). Stained leaf disks were observed using an optical microscope at magnification x40. Sterilized distilled water was used for mock treatment. Scale bar = 50 um.

To investigate features of blister-like lesions on *N*. *benthamiana* leaves after infiltration with *C*. *michiganensis* LMG7333^T^ and *C*. *capsici* PF008^T^, leaf disks from infiltrated *N. benthamiana* leaves were collected and treated with trypan blue solution 0, 1, 3, 5, and 7 dai (Figure 2). Dead cells turned to blue after staining, as trypan blue is normally used for observation of such cells. Blue color spots began to appear from 3 dai, and the number and size of those spots were gradually increased (Figure 2). These patterns were exactly matched with blister-like lesions, indicating that plant cells in blister-like lesions are undergoing death.

Generally, ROS generation is accompanied by cell death (Balint-Kurti, 2019). Thus, the 3,3′-diaminobenzidine (DAB) staining was conducted with leaf disks collected from *N*. *benthamiana* leaves after infiltration with two *Clavibacter* species (Supplementary Figure S1). The stained spots by DAB began to appear from 3 dai, and the intensity gradually increased (Supplementary Figure S1). Like trypan blue staining, DAB-stained spots exactly matched with the areas of blister-like lesions, indicating that blister-like lesions are generated by cell death, and accompanied by the generation of ROS, such as hydroxy peroxide.

### Two Important Virulence Genes of *C. michiganensis* in Tomatoes Are Not Required for the Development of Blister-Like Lesions and Rapid Necrosis on *N. benthamiana* Leaves

**Figure 3:**
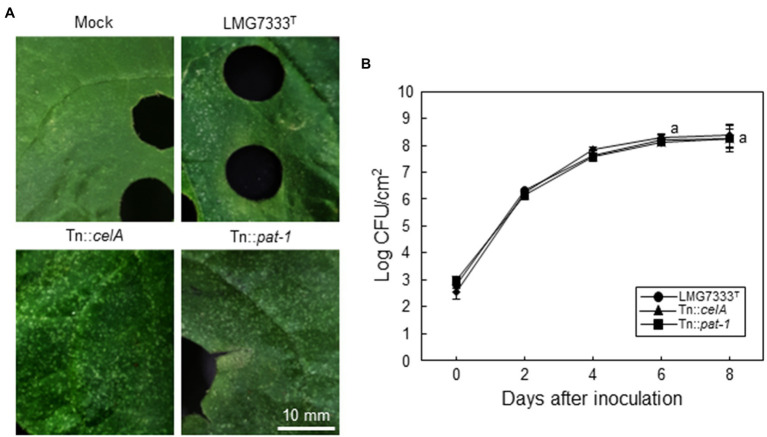
No contribution of two major virulence genes of *C*. *michiganensis* to the development of blister-like symptoms on *N*. *benthamiana* leaves. **(A)** Blister-like symptoms on *N*. *benthamiana* leaves by infiltration of 5×10^4^ CFU/ml *C*. *michiganensis* LMG7333^T^ and its Tn::*celA* or Tn::*pat-1* mutant strains. The leaves were photographed at 8 days after inoculation (dai). **(B)** The bacterial growth of *C*. *michiganensis* LMG7333^T^, Tn::*celA*, and Tn::*pat-1* strains in *N*. *benthamiana* leaves at the indicated time points. An average and standard deviation (*n* = 3) of each bacterial titer are shown in the figure. The letters at 6 and 8 dai in the graphs indicate a statistically significant difference analyzed *via* Duncan’s multiple range test (*p* < 0.05). Similar results were obtained from three independent assays. Scale bar = 10 mm.

Previously, it was shown that *celA* and *pat-1* genes of *C*. *michiganensis* are critical for disease development in tomatoes (Gartemann et al., 2003; Hwang et al., 2019). Thus, we tried to determine whether these two important virulence genes are required for the development of blister-like lesions on *N*. *benthamiana* leaves. For this, Tn::*celA* and Tn::*pat-1* mutant strains of *C*. *michiganensis* LMG7333^T^ were infiltrated into *N*. *benthamiana* leaves, and the formation of blister-like lesions was monitored. Intriguingly, when 5×10^4^ CFU/ml of each mutant strain was infiltrated, each caused as many blister-like lesions on leaves as the WT strain (Figure 3A). Moreover, those mutant strains grew as much as the WT strain (Figure 3B). These results indicate that two known important virulence genes are not required for the formation of blister-like lesions in *N. benthamiana*.

**Figure 4:**
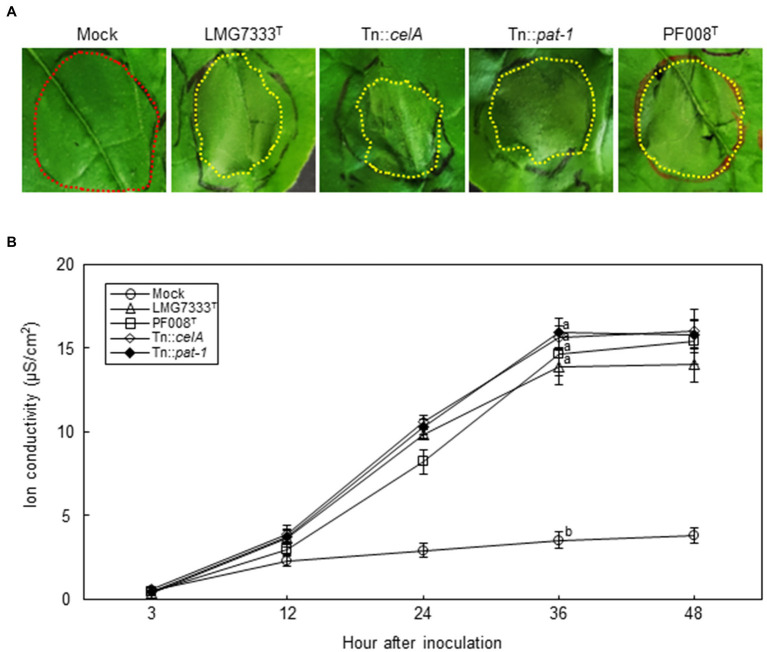
Influence of two major virulence genes of *C*. *michiganensis* for necrosis on *N*. *benthamiana* leaves. **(A)** The necrosis symptoms on *N*. *benthamiana* leaves by infiltration with 10^8^ CFU/ml of *C*. *capsici* PF008^T^, *C*. *michiganensis* LMG7333^T^ and its mutant strains. Each red and yellow dotted line indicates no symptom and necrotic parts, respectively. Photos showing necrosis symptoms were taken at 48 hours after infiltration (hai), respectively. **(B)** The measurement of ion conductivity on *N*. *benthamiana* leaves after inoculation with 10^8^ CFU/ml of the bacterial strains. The letters at the time points in the graphs indicate a statistically significant difference analyzed *via* Duncan’s multiple range test (*p* < 0.05). Similar results were obtained from two independent assays. Mock, 10 mM MgCl_2_.

To examine whether different bacterial concentrations cause different symptoms, *N*. *benthamiana* leaves were infiltrated with a higher bacterial titer (10^8^ CFU/ml) of *C*. *michiganensis* LMG7333^T^ or *C*. *capsici* PF008^T^. In this condition, the infiltrated regions by both bacterial pathogens rapidly displayed water-soaking symptoms, followed by typical necrosis by 48 h after inoculation (hai) (Figure 4A). Ion conductivity was measured in infiltrated *N*. *benthamiana* leaves to determine the speed of necrosis onset. The ion conductivity began to significantly increase from 12 hai and continuously increased until 36 hai in *N*. *benthamiana* leaves infiltrated with both *Clavibacter* species (Figure 4B). These results indicate that infiltration of *N. benthamiana* leaves with a high concentration of *Clavibacter* species causes rapid necrosis without blister-like lesions.

Next, to examine whether *celA* and *pat-1* genes are required for the development of necrosis, 10^8^ CFU/ml of Tn::*celA* and Tn::*pat-1* mutant strains were infiltrated into *N. benthamiana* leaves. Results showed that these mutants caused necrosis like the WT strain (Figure 4A) and increased ion conductivity as much as the WT (Figure 4B), indicating that these two virulence genes in tomatoes are not critical for necrosis in *N. benthamiana*.

### *C. michiganensis*, but Not *C. capsici*, Caused Wilting on *N. benthamiana*

**Figure 5:**
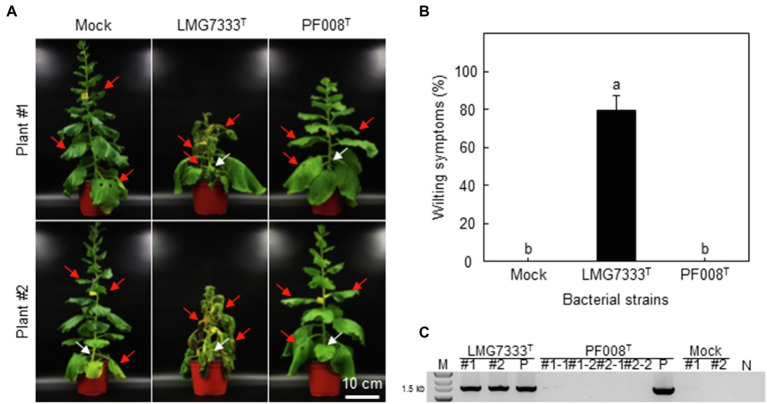
Wilting symptoms caused after stem inoculation with *C*. *michiganensis* LMG7333^T^, but not after *C*. *capsici* PF008^T^ inoculation in *N*. *benthamiana* plants. **(A)** Wilting symptoms caused in *N*. *benthamiana* plants after injection with 20 ul of 10^8^ CFU/ml of *C*. *michiganensis* LMG7333^T^ and *C*. *capsici* PF008^T^. Injection sites in stems are indicated by white arrows. The inoculated plants were photographed at 21 days after inoculation (dai). **(B)** Quantification of wilting severity in *N*. *benthamiana* plants shown in **(A)**. An average and standard deviation (*n* = 4) of wilting severity were obtained from two independent assays. The different letters on top of each bar indicate a statistically significant difference analyzed *via* Kruskal–Wallis test (*p* < 0.05). **(C)** Identification of inoculated bacteria in two wilted *N*. *benthamiana* plants (#1 and #2) *via* PCR analysis. The leaf disks (1 cm in diameter) were collected at three leaves (indicated by red arrows) from the inoculated plants at 21 dai. M, 1 kb DNA marker; P, bacterial cells of each *Clavibacter* species as positive control; N, no DNA. Scale bar = 10 cm.

*C. michiganensis* causes not only blister-like lesions, but also wilting and canker symptoms in tomatoes (de León et al., 2011). To examine whether both *C*. *michiganensis* LMG7333^T^ and *C*. *capsici* PF008^T^ cause wilting in *N*. *benthamiana*, 10^8^ CFU/ml bacterial suspension of two *Clavibacter* species was injected into the main stems *via* the stem inoculation method, and wilting development was monitored. Notably, *C*. *michiganensis* LMG7333^T^ caused severe wilting in *N*. *benthamiana*, as in tomatoes, whereas *C*. *capsici* PF008^T^ did not (Figure 5A). Wilting symptoms began to develop on the unilateral side of inoculation sites in *N*. *benthamiana*. After 3 weeks, severe wilting and necrosis were observed on the whole *N*. *benthamiana* plant only by *C*. *michiganensis* LMG7333^T^ (Figure 5B).

Another main disease symptom of *Clavibacter* species in host plants is bacterial canker. Bacterial canker development on *N*. *benthamiana* stems by *C*. *michiganensis* infection has been previously reported (Balaji et al., 2011). Consistently, in this experiment, both *C*. *michiganensis* LMG7333^T^ and *C*. *capsici* PF008^T^ caused significant bacterial canker on inoculated *N*. *benthamiana* stems (Supplementary Figure S2).

To confirm that wilting was caused by *C. michiganensis* bacterium, the polymerase chain reaction (PCR) test was conducted for identification of this bacterium using leaf samples that were taken from locations near and above the inoculation site. PCR results showed that *C*. *michiganensis* LMG7333^T^ could move to the entire plant, likely through its xylem vessels, whereas *C*. *capsici* PF008^T^ could not move to nearby leaves nor in an upper direction in *N*. *benthamiana* (Figure 5C).

*Clavibacter* species normally invade the host plants through wounds and natural openings (Carlton et al., 1998; de León et al., 2011; Tancos et al., 2013). Thus, we sprayed a bacterial suspension of both *Clavibacter* species onto the *N*. *benthamiana* plants to mimic the natural invasion process. However, neither bacterial species caused any visible symptoms (Supplementary Figure S3A). PCR results also showed no evidence of the bacterial presence of both *Clavibacter* species inside plants (Supplementary Figure S3B). These results indicate that even *C*. *michiganensis* LMG7333^T^ fails to actively infect *N. benthamiana* through stomata.

### Two Important Virulence Genes of *C.* *michiganensis* in Tomatoes Are Partially Required for the Development of Wilting on *N. benthamiana*

**Figure 6:**
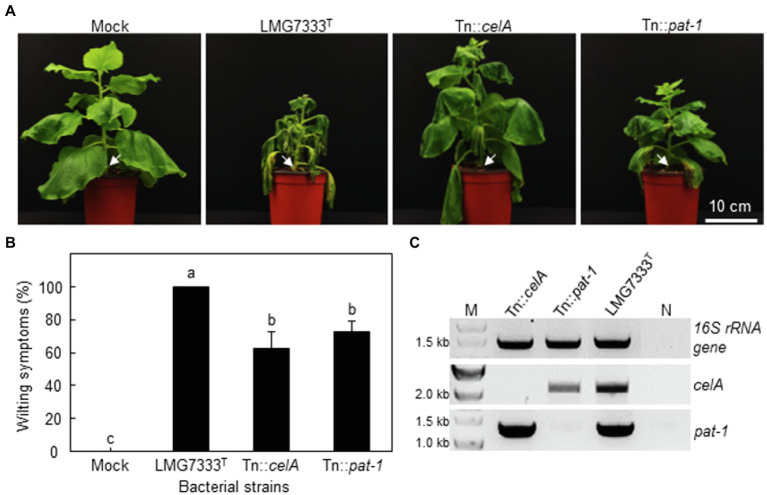
Influence of two major virulence genes of *C*. *michiganensis* in *N*. *benthamiana* plants on the development of wilting symptoms. **(A)** Wilting symptoms in *N*. *benthamiana* after stem inoculation with 10^8^ CFU/ml of *C*. *michiganensis* LMG7333^T^ and its Tn::*celA* and Tn::*pat-1* mutant strains. Inoculation sites are indicated by white arrows. The inoculated plants were photographed at 21 days after inoculation (dai). **(B)** Quantification of wilting severity in *N*. *benthamiana* plants shown in (A). An average and standard deviation (*n* = 4) of wilting severity are shown in the figures. The different letters on top of each bar indicate a statistically significant difference analyzed *via* Kruskal–Wallis test (*p* < 0.05). Similar results were obtained from three independent assays. **(C)** Confirmation of inoculated bacteria *via* PCR analysis with primer sets specific to the indicated genes. M, 1 kb DNA marker; N, no DNA. Scale bar = 10 cm.

To examine whether *celA* and *pat-1* genes are required for the development of wilting in the *N*. *benthamiana* plant, 10^8^ CFU/ml of *C*. *michiganensis* WT, Tn::*celA* and Tn::*pat-1* mutant strains were inoculated using the stem inoculation method, and the development of wilting was monitored. Both mutants caused delayed wilting in *N. benthamiana* (Figure 6A), and the wilting severity was approximately 70% of that caused by WT *C*. *michiganensis* (Figure 6B). The presence of mutant strains in wilting *N. benthamiana* plants was confirmed *via* PCR test (Figure 6C). When *N. benthamiana* was inoculated using the root-dipping method with 10^9^ CFU/ml of the bacterial suspensions, wilting results were similar to those of the stem inoculation method (Supplementary Figure S4). These results indicate that, like tomatoes, *celA* and *pat-1* genes are partially required for wilting development in *N. benthamiana*.

### HR-Like Cell Death Was Induced on *N. tabacum* Leaves by *Clavibacter* Species

**Figure 7:**
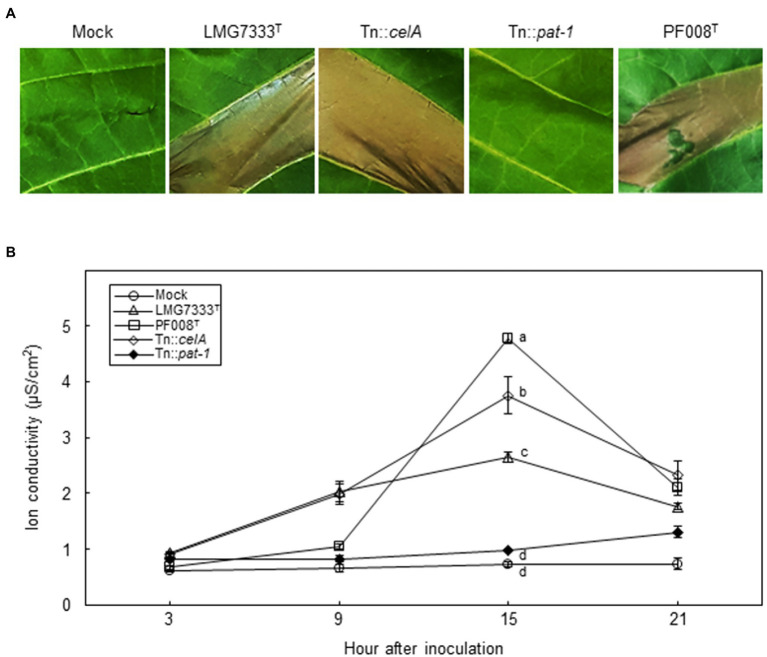
Influence of two major virulence genes of *C*. *michiganensis* for HR-like cell death on *N*. *tabacum* leaves. **(A)** The HR-like cell death on *N*. *tabacum* leaves by infiltration with 10^8^ CFU/ml of *C*. *capsici* PF008^T^, *C*. *michiganensis* LMG7333^T^, and its mutant strains. Photos showing HR-like cell death were taken at 18 h after infiltration (hai), respectively. **(B)** The measurement of ion conductivity on *N*. *tabacum* leaves after inoculation with 10^8^ CFU/ml of the bacterial strains. The letters at the time points in the graphs indicate a statistically significant difference analyzed *via* Duncan’s multiple range test (*p* < 0.05). Similar results were obtained from two independent assays. Mock, 10 mM MgCl_2_.

We showed that no disease symptoms were observed after infiltration of a low bacterial concentration (5×10^4^ CFU/ml) of two *Clavibacter* species into *N. tabacum* leaves (Figure 1). To examine whether these *Clavibacter* species can induce HR-like cell death in *N. tabacum*, 10^8^ CFU/ml of *C. capsici* PF008^T^ and *C. michiganensis* LMG7333^T^ were infiltrated. Results showed that both induced HR-like cell death within 18 hai (Figure 7A), and ion conductivity began to increase from 9 hai until 15 hai (Figure 7B). These results indicate that both *Clavibacter* species can induce HR-like cell death in *N. tabacum*, presenting another clue that *N*. *tabacum* is a non-host plant of these bacteria.

To determine whether *celA* and *pat-1* genes are required for induction of HR-like cell death, mutant strains were infiltrated. The Tn::*celA* mutant induced HR-like cell death like the WT strain, whereas the Tn::*pat-1* mutant failed (Figure 7A). The ion conductivity was consistent with these phenotypes (Figure 7B). These results indicate that the *pat-1* gene, but not *celA*, is critical for HR induction in *N. tabacum*.

## Discussion

In this study, we show that *N. benthamiana* displays blister-like lesions and rapid necrosis in leaves after infection with Gram-positive and plant-pathogenic bacteria *C. michiganensis* and *C. capsici* and wilting after infection with *C. michiganensis* (Table 1). On the basis of these results and as reported previously (Balaji et al., 2011), we propose that *N. benthamiana* is a surrogate host plant of *Clavibacter* pathogens, particularly, *C. michiganensis*. *N. benthamiana* as well as *N. tabacum* have been widely used as model plants for diverse research on plant–microbe interactions (Goodin et al., 2008). Unlike *N. benthamiana*, *N. tabacum* appears to be a non-host plant of *Clavibacter* pathogens because those pathogens induced HR-like cell death and grew much less in *N. tabacum* than in *N. benthamiana*.

**Table 1:** Summary of phenotypes in *Nicotiana* plants with *Clavibacter* strains in this study.

			Bacterial strains			
*Nicotiana* species	Inoculum concentration (CFU/ml)	Inoculation site	*C*. *michiganensis* LMG7333^T^	*C*. *michiganensis* Tn::*celA*	*C*. *michiganensis* Tn::*pat-1*	*C*. *capsici* PF008^T^
*N*. *benthamiana*	5×10^4^	Leaf	Blister-like lesions	Blister-like lesions	Blister-like lesions	Blister-like lesions
	10^8^	Leaf	Necrosis	Necrosis	Necrosis	Necrosis
		Stem	Systemic wilting	Systemic wilting	Systemic wilting	No wilting
	10^9^	Root	Systemic wilting	Systemic wilting	Systemic wilting	ND^*^
*N*. *tabacum*	5×10^4^	Leaf	No symptoms	ND	ND	No symptoms
	10^8^	Leaf	HR	HR	No HR	HR

* *ND, not determined.*

The *celA* and *pat-1* genes of *C. michiganensis*, encoding a cellulase and a putative serine protease as apoplastic effectors, are critical in the development of disease symptoms in tomatoes (Gartemann et al., 2003; Hwang et al., 2019). However, these genes were unnecessary for the formation of blister-like lesions and rapid necrosis in *N. benthamiana* leaves (Figures 3, 4) and were partially necessary for complete wilting (Figure 6; Supplementary Figure S4). Previously, it was shown that *C. michiganensis* strain *Cmm*100, which lacks plasmids pCM1 and pCM2, formed blisters in tomato leaves (Chalupowicz et al., 2017); *celA* and *pat-1* genes are located in pCM1 and pCM2, respectively (Dreier et al., 1997; Jahr et al., 2000). Moreover, *C. capsici* does not have *celA* but carries the *pat-1* ortholog. However, this *pat-1* ortholog failed to complement the pathogenicity function of the *C. michiganensis pat-1* gene in tomatoes, whereas another *pat-1* ortholog, *chp-7* of *C. sepedonicus*, could partially complement it (Hwang et al., 2022). Although both Pat-1 and Chp-7 could elicit HR in *N. tabacum* (Nissinen et al., 2009; Lu et al., 2015), *chp-7* failed to complement HR-eliciting ability of the Tn::*pat-1* mutant of *C. michiganensis* (Hwang et al., 2022), indicating that these orthologs might use different mechanisms for HR elicitation in *N. tabacum*. Nevertheless, *C. michiganensis* caused blister-like lesions and rapid necrosis in *N. benthamiana* leaves (Figures 1, 4). These results collectively imply that *celA* and *pat-1* genes are not connected with the formation of blister-like lesions. These findings reveal that novel pathogenicity or virulence factors of *C. michiganensis* are minimally required for the formation of blister-like lesions and rapid necrosis in leaves, and more virulence factors are necessary for complete wilting. Notably, the *chpC* gene in the chromosomal PAI region of *C. michiganensis* and other genes, such as *stbA*, *pgaA*, and *endX/Y*, appears to contribute to the formation of blisters in tomato leaves (Chalupowicz et al., 2017). Revealing the novel pathogenicity or virulence factors of *C. michiganensis* for the formation of blister-like lesions and rapid necrosis in leaves will help us understand the virulence mechanisms of this bacterium in *N. benthamiana*. Based on previous literature (Jacques et al., 2012; Tancos et al., 2015; Thapa et al., 2017), variation in the virulence of *C. michiganensis* natural isolates exists in tomato. It will be worthwhile to examine whether there are natural isolates of *C. michiganensis* showing different virulence patterns in tomato and *N. benthamiana*.

*C*. *michiganensis* caused severe wilting in *N*. *benthamiana*, whereas *C. capsici* did not (Figure 5). *C. capsici* causes bacterial canker in pepper stems without wilting symptom (Hwang et al., 2018), consistent with no wilting development in *N. benthamiana*. Previously, we showed that the introduction of *celA* into *C. capsici* resulted in increased ability to cause wilting in tomatoes (Hwang et al., 2018). However, the wilting severity was much less than that caused by *C. michiganensis*, implying that more factors, which might be missing in *C. capsici*, are required for severe wilting. It will be useful to study *N. benthamiana* plants to reveal those factors using both *Clavibacter* species.

Although blisters were shown in tomato leaves after infection with *C. michiganensis* (Chalupowicz et al., 2017), its features have not been studied. We showed that blister-like lesions caused by two *Clavibacter* species in *N. benthamiana* leaves were closely associated with cell death and the generation of ROS (Figure 2; Supplementary Figure S1). The color of blisters in tomatoes and the blister-like lesions in *N. benthamiana* leaves appear pale green, indicating that chlorophyll might be degraded during blister formation, and infected cells may eventually die. ROS might be responsible for these processes. It will be worthwhile to determine how blister-like lesions are formed in *N. benthamiana* leaves, which will help us understand the formation of blisters in tomato leaves.

Taken together, our results suggest that *N*. *benthamiana* is a surrogate host of *C*. *michiganensis* and *C. capsici* and *N*. *tabacum* is a non-host plant of both *Clavibacter* species. Although disease phenotypes in *N. benthamiana* after inoculation with two *Clavibacter* species appear very similar to those in natural host plants, such as tomato and pepper, different virulence factors might be necessary. Therefore, *N. benthamiana* can be used to understand the novel molecular mechanisms of *Clavibacter* pathogens for virulence or pathogenicity in the future.

## Data Availability Statement

The original contributions presented in the study are included in the article/Supplementary Material, and further inquiries can be directed to the corresponding author.

## Author Contributions

IP, IH, E-JO, C-TK, and C-SO conducted experiments, analyzed the data, and wrote the manuscript. All authors contributed to the article and approved the submitted version.

## Funding

This work was supported by the National Research Foundation of Korea (NRF) grant funded by the Korean government (MIST; 2019R1A2C2004568 and 2018R1A5A1023599).

## Conflict of Interest

The authors declare that the research was conducted in the absence of any commercial or financial relationships that could be construed as a potential conflict of interest.

## Publisher’s Note

All claims expressed in this article are solely those of the authors and do not necessarily represent those of their affiliated organizations, or those of the publisher, the editors and the reviewers. Any product that may be evaluated in this article, or claim that may be made by its manufacturer, is not guaranteed or endorsed by the publisher.

## Supplementary Material

The Supplementary Material for this article can be found online at: https://www.frontiersin.org/articles/10.3389/fpls.2022.876971/full#supplementary-material

Supplementary Figure S1 3,3′-Diaminobenzidine (DAB) straining of *N*. *benthamiana* leaves showing blister-like symptoms, after infiltration with 5×10^4^ CFU/ml bacterial suspensions of *C*. *michiganensis* LMG7333^T^ and *C*. *capsici* PF008^T^.

Supplementary Figure S2 Canker development on *N. benthamiana* stems *via* the stem inoculation with 10^8^ CFU/ml bacterial suspensions of *C*. *michiganensis* LMG7333^T^, *C*. *capsici* PF008^T^, and *C*. *michiganensis* LMG7333 Tn::*celA* and Tn::*pat-1* mutant strains.

Supplementary Figure S3 No symptoms after spray inoculation with *C*. *michiganensis* LMG7333^T^ and *C*. *capsici* PF008^T^ in *N*. *benthamiana*.

Supplementary Figure S4 Influence of two major virulence genes of *C. michiganensis* in *N. benthamiana* plants for the development of wilting symptoms.
