# Supplementary material for: Nicotianabenthamiana, a Surrogate Host to Study Novel Virulence Mechanisms of Gram-Positive Bacteria, Clavibacter michiganensis, and C. capsici in Plants
Source: Front Plant Sci. 2022 May 10;13:876971. doi: 10.3389/fpls.2022.876971 (PMC9127732; doi:10.3389/fpls.2022.876971)
Supplement: Supplementary Figure S1 — 3,3′-Diaminobenzidine (DAB) straining of N. benthamiana leaves showing blister-like symptoms, after infiltration with 5×10^4^ CFU/ml bacterial suspensions of C. michiganensis LMG7333T and C. capsici PF008T. [file Table_1.DOCX]

Supplementary Material


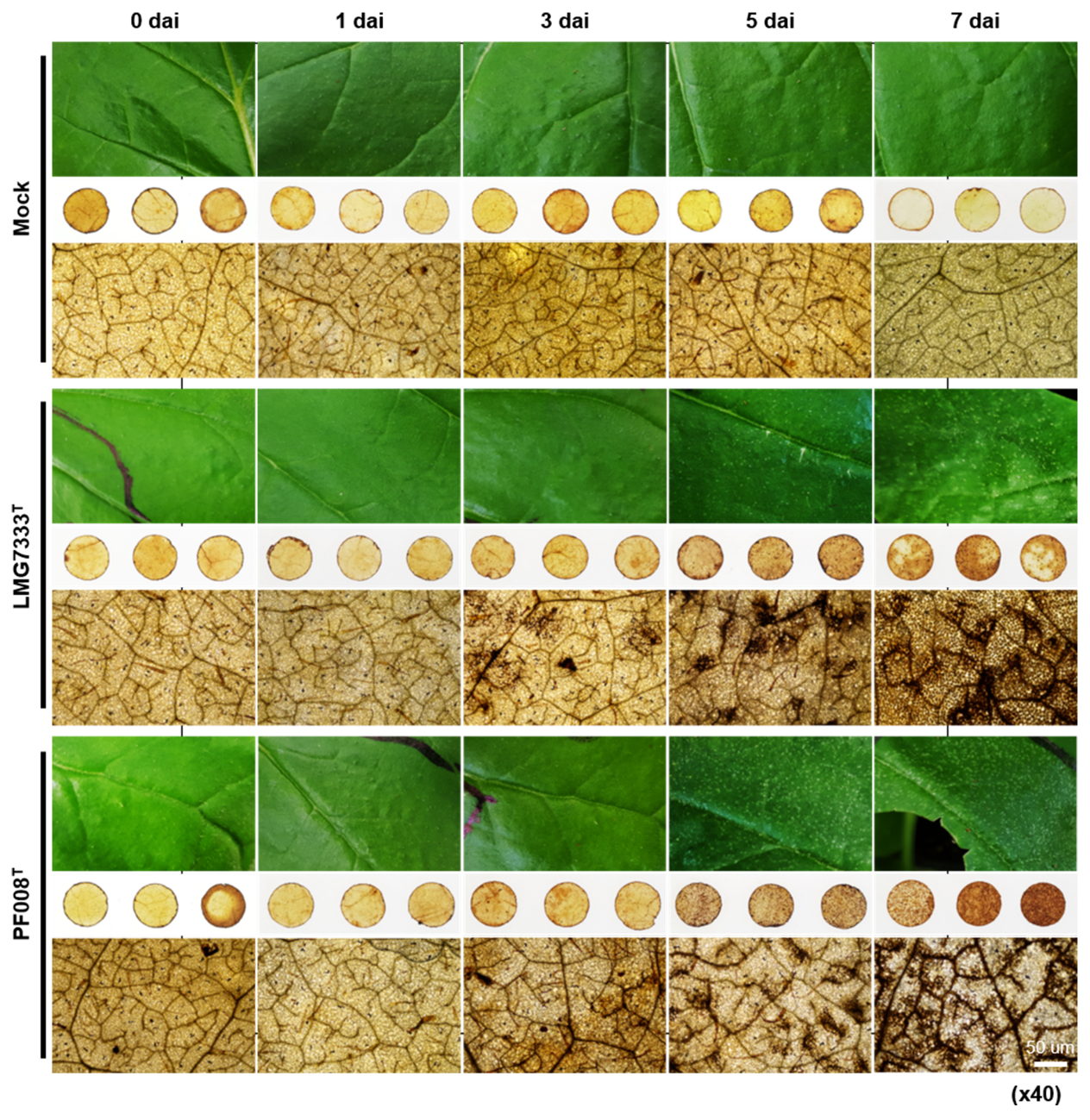


**Supplementary Figure S1.** 3,3′-diaminobenzidine (DAB) straining of *N*. *benthamiana* leaves showing blister-like symptoms, after infiltration with 5x10^4^ CFU/ml bacterial suspensions of *C*. *michiganensis* LMG7333^T^ and *C*. *capsici* PF008^T^. Three leaf disks (1cm in diameter) per treatment were collected for DAB staining at 0, 1, 3, 5 and 7 days after inoculation (dai). Stained leaf disks were observed by an optical microscope at magnification x40. Sterilized distilled water was used for mock treatment. Scale bar = 50 um.


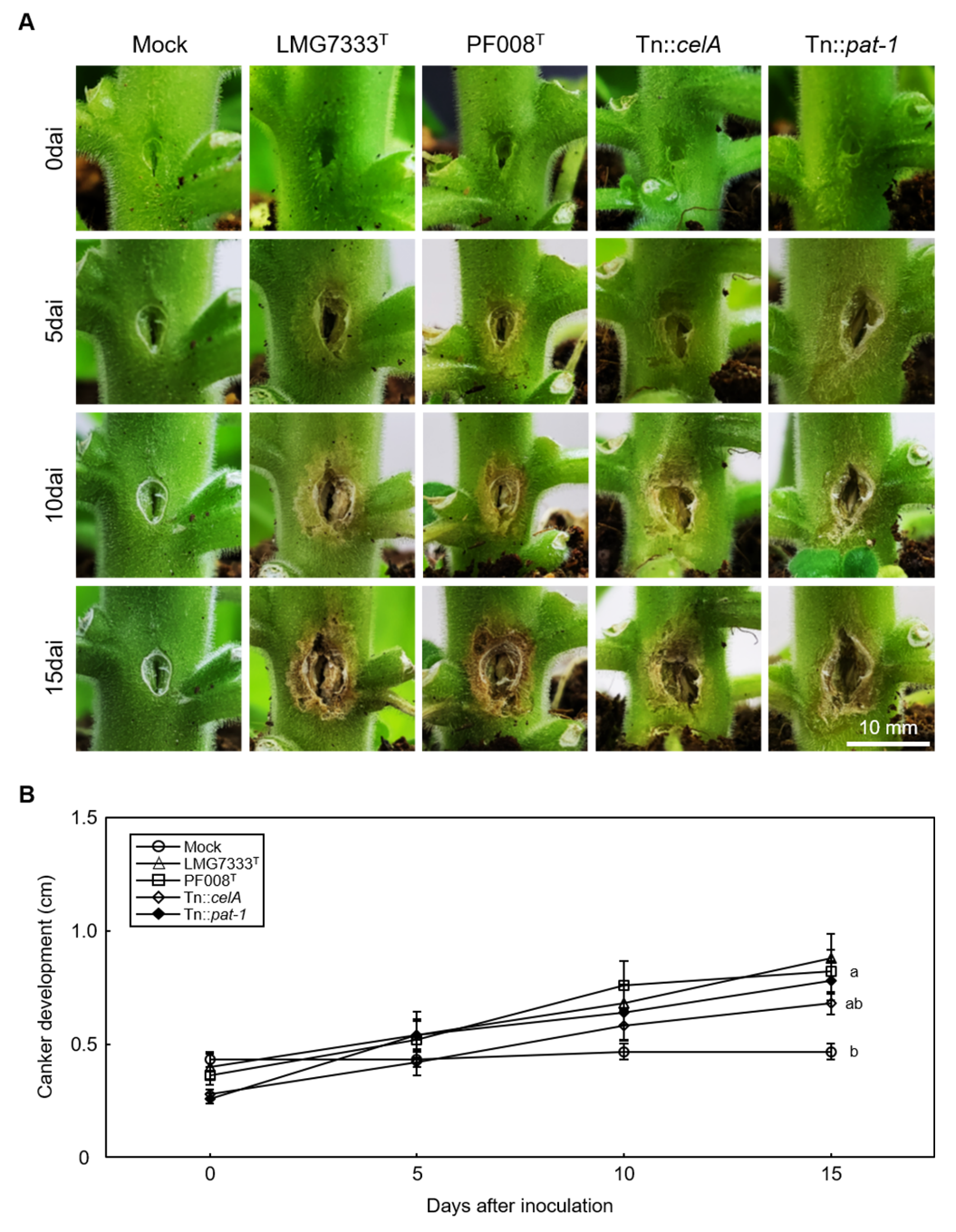


**Supplementary Figure S2.** Canker development on *N. benthamiana* stems via stem inoculation with 10^8^ CFU/ml bacterial suspensions of *C*. *michiganensis* LMG7333^T^, *C*. *capsici* PF008^T^, and *C*. *michiganensis* LMG7333 Tn::*celA* and Tn::*pat-1* mutant strains. **(A)** Development of bacterial canker symptoms from inoculation sites on *N. benthamiana* stems. The symptoms were photographed at 0, 5, 10, and 15 days after inoculation (dai). **(B)** The measurement of canker sizes. An average and standard error (n = 5) of each canker size are shown in the figure. The letters at the time points in the graphs indicate a statistically significant difference analyzed via Duncan’s multiple range test (*p* < 0.05). Scale bar = 10 mm.


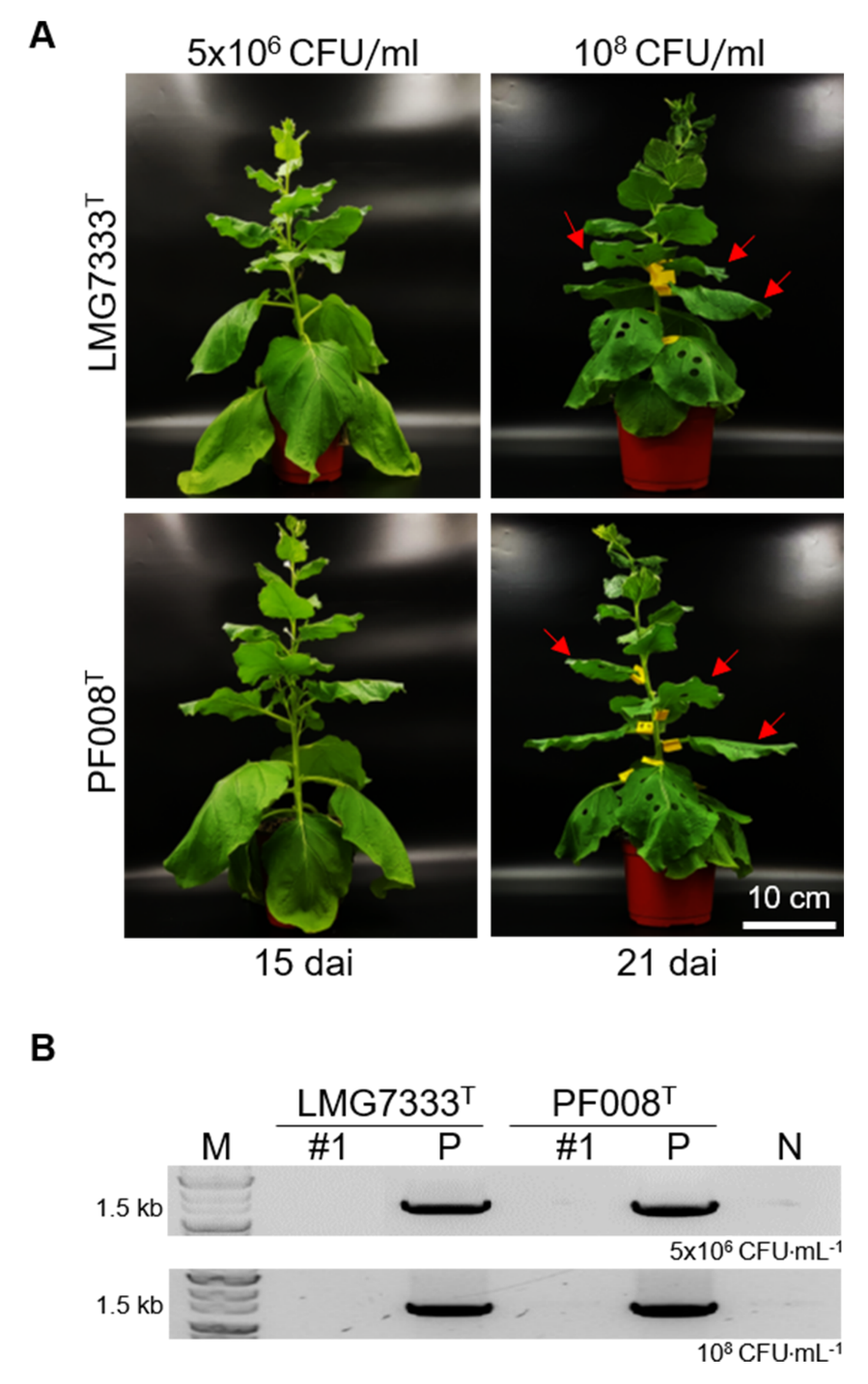


**Supplementary Figure S3.** No symptoms after spray inoculation with *C*. *michiganensis* LMG7333^T^ and *C*. *capsici* PF008^T^ in *N*. *benthamiana*. **(A)** *N*. *benthamiana* plants via spray inoculation with 5x10^6^ CFU/ml or 10^8^ CFU/ml bacterial suspensions of *C*. *michiganensis* LMG7333^T^ and *C*. *capsici* PF008^T^. Red arrows indicate locations where samples were collected for PCR analysis in **(B)**. The inoculated plants were photographed at 15 and 21 days after inoculation (dai). Three plants were used per treatment, and similar results were obtained from three independent assays. **(B)** Identification of inoculated bacteria via PCR analysis in the inoculated *N*. *benthamiana* plants. The leaf disks (1cm in diameter) were collected at three leaves indicated by red arrows from the inoculated plants (#1). M, 1kb DNA marker; P, bacterial cells of each *Clavibacter* species as positive control; N, no DNA. Scale bar = 10 cm.


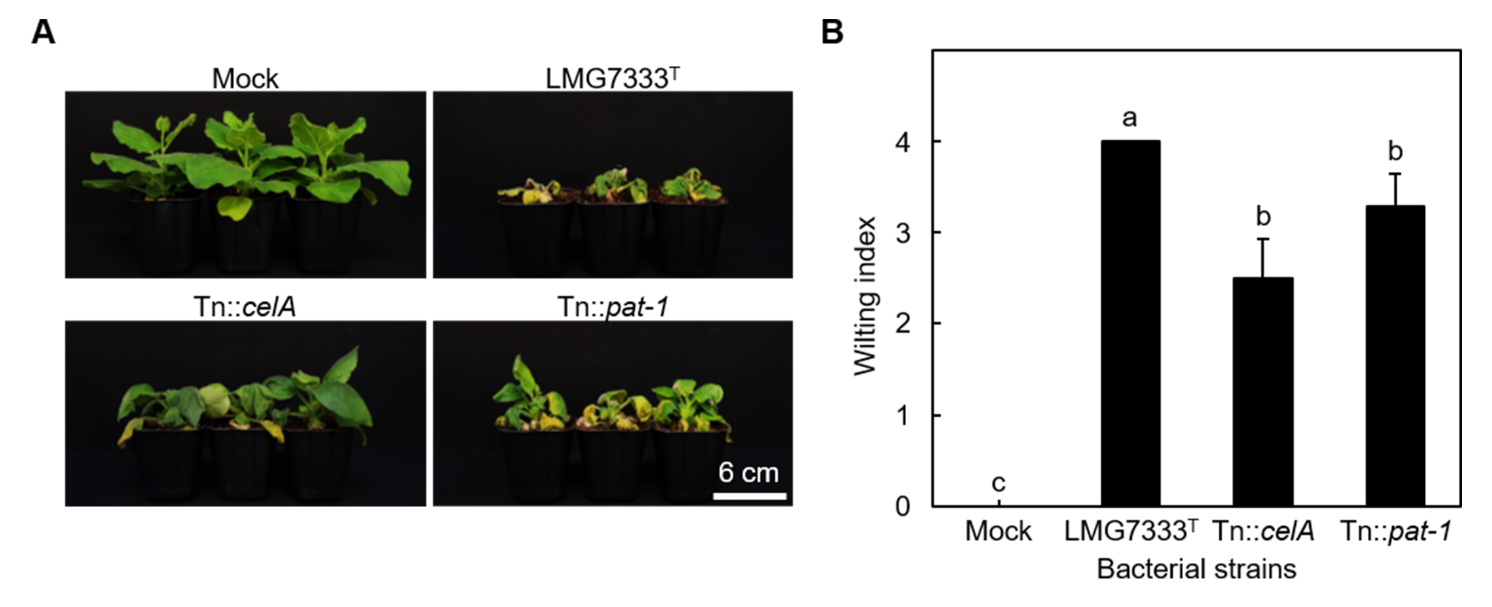


**Supplementary Figure S4.** Influence of two major virulence genes of *C. michiganensis* in *N. benthamiana* plants on the development of wilting symptoms. **(A)** Wilting symptoms in *N*. *benthamiana* after root-dip inoculation with 10^9^ CFU/ml of *C*. *michiganensis* LMG7333^T^ and its Tn::*celA* and Tn::*pat-1* mutant strains. The inoculated plants were photographed at 18 days after inoculation (dai). **(B)** Quantification of the wilting severity in *N*. *benthamiana* plants shown in **(A)**. An average and standard deviation (n = 4) of wilting severity are shown in the figures. The different letters on top of each bar indicate a statistically significant difference analyzed via Kruskal-Wallis test (*p* < 0.05). Similar results were obtained from three independent assays. Scale bar = 6 cm.
